# Supplementary material for: Short-Term Safety and Psychosocial Impact of the BNT162b2 mRNA COVID-19 Vaccine in Cancer Patients—An Italian Single-Center Experience
Source: Biomedicines. 2023 Jan 9;11(1):165. doi: 10.3390/biomedicines11010165 (PMC9855735; doi:10.3390/biomedicines11010165)
Supplement: Supplementary file 1 [file biomedicines-11-00165-s001.zip › biomedicines-2090430-supplementary.pdf]

Psychological impact and side effects/adverse events after the first dose of BNT162b2 Sars-CoV-2 vaccine

The following *ad hoc* clinical-generated anonymous questionnaire is administered to patients who received SARS-CoV-2 vaccine (BNT162b2) at the Department of Oncology of San Luigi Gonzaga University Hospital, from 31st March 2021 to 19th April 2021.

The questionnaire is composed by two parts: the first is dedicated to patient's oncological history and may be compiled with the assistance of a medical doctor during the clinical examination before vaccination. The second part is focused on the psychological and social impact of vaccination and side effects/adverse events occurred after the first dose of BNT162b2, SARS-CoV-2 vaccine.

By compiling the questionnaire the patient accepts to be questioned about the side effects/adverse events experienced after the second dose via telephone questionnaire that will be administered 4-7 days after.

By compiling the anonymous questionnaire, the patient attests to have read and understood the information above and gives the informed consent for this observational study.

All data collected will be kept confidential in compliance with the current regulations.

1st PART: Oncological history

Type of malignancy	
Thoracic (lung or pleural)	
Gastrointestinal	
Prostate	
Breast	
Genitourinary	
Endocrine	
Gynaecological	
Others	

Therapy	
Chemotherapy (ChT)	
Immune-checkpoint inhibitors (ICIs)	
ChT-ICIs	
Other systemic therapies	
Targeted therapy	
Hormone therapy	
Locoregional treatment	
Newly diagnosed patient	
Follow-up	
**Stage disease**	
Early-stage	
Advanced disease	
Disease-free	

2nd PART: Patient's section

General information		
**Age**		
**Sex**	M	F
**SARS-CoV-2 infection history**		
**Have you ever been infected by SARS-CoV-2?**	Yes	No
**If yes, please specify the period when the infection occurred**:<br>• From March 2020 to May 2020;<br>• From June 2020 to September 2020;<br>• From October 2020 to January 2021.		
**If yes, specify the health-care assistance you have received:**<br>• None, an asymptomatic infection occurred;<br>• Home care assistance;<br>• Hospitalization (low intensity of care);<br>• Hospitalization (intensive care unit).		
**Vaccine**		
**Before receiving the first dose of BNT162b2 SARS-CoV-2 vaccine, you felt a sense of:**		
Fear	Yes	No
Insecurity	Yes	No
Indifference	Yes	No
Confidence	Yes	No
Enthusiasm	Yes	No
**After receiving the first dose of BNT162b2 SARS-CoV-2 vaccine, you felt a sense of:**		
Fear	Yes	No
Insecurity	Yes	No
Indifference	Yes	No
Confidence	Yes	No
Enthusiasm	Yes	No
**After receiving BNT162b2 SARS-CoV-2 vaccine you feel:**		
More confident in visiting public places (i.e. cinemas, malls)	Yes	No
More confident in spending time with your family/friends	Yes	No
More confident in attending check-up visits	Yes	No
More confident in practising recreational and sport activities	Yes	No
**You consider information you have received about SARS-CoV-2 vaccine prior to recruitment in the vaccine campaign:**		
Insufficient	Yes	No
Confused	Yes	No
Adequate	Yes	No

**Your own opinion about SARS-CoV-2 vaccine has been mostly influenced by:**		
• General practitioner/oncologist or other specialists;<br>• Family/friends;<br>• Mass-media;<br>• Scientific literature.		
**Would you have accepted to be vaccinated if you were proposed a different serum?**	Yes	No
**Patient's allergy history**		
**Do you suffer from any form of allergy (any food or drugs)?**	Yes	No
**Have you ever received an allergological examination?**	Yes	No
**If yes, have you been prescribed an anti-allergic prophylaxis before receiving the vaccine?**	Yes	No
**Have you been recommended to be clinically observed more than fifteen minutes after receiving the vaccine?**	Yes	No
**Side effects/adverse events**		
**Have you experienced any side effect/adverse event after the first dose of BNT162b2 SARS-CoV-2 vaccine?**	Yes	No
**If yes, please specify the type of side effect/adverse event**		
Injection site pain/rash	Yes	No
Diffuse joints pain	Yes	No
Fever	Yes	No
Chills or night sweating	Yes	No
Headache	Yes	No
Nausea, vomit or diarrhea	Yes	No
Fatigue	Yes	No
Insomnia	Yes	No
Lymph nodes enlargement	Yes	No
Mild allergic reaction (rash, diffuse pruritus)	Yes	No
Severe allergic reaction (dyspnoea, anaphylaxis)	Yes	No
